# Folding and binding pathways of BH3-only proteins are encoded within their intrinsically disordered sequence, not templated by partner proteins

**DOI:** 10.1074/jbc.RA118.002791

**Published:** 2018-05-01

**Authors:** Michael D. Crabtree, Carolina A. T. F. Mendonça, Quenton R. Bubb, Jane Clarke

**Affiliations:** From the Department of Chemistry, University of Cambridge, Cambridge CB2 1EW, United Kingdom

**Keywords:** apoptosis, B cell lymphoma 2 (Bcl-2) family, biophysics, kinetics, protein folding, protein–protein interaction, thermodynamics, coupled folding and binding, IDP, φ value

## Abstract

Intrinsically disordered regions are present in one-third of eukaryotic proteins and are overrepresented in cellular processes such as signaling, suggesting that intrinsically disordered proteins (IDPs) may have a functional advantage over folded proteins. Upon interacting with a partner macromolecule, a subset of IDPs can fold and bind to form a well-defined three-dimensional conformation. For example, disordered BH3-only proteins bind promiscuously to a large number of homologous BCL-2 family proteins, where they fold to a helical structure in a groove on the BCL-2–like protein surface. As two protein chains are involved in the folding reaction, and the structure is only formed in the presence of the partner macromolecule, this raises the question of where the folding information is encoded. Here, we examine these coupled folding and binding reactions to determine which component determines the folding and binding pathway. Using Φ value analysis to compare transition state interactions between the disordered BH3-only proteins PUMA and BID and the folded BCL-2–like proteins A1 and MCL-1, we found that, even though the BH3-only protein is disordered in isolation and requires a stabilizing partner to fold, its folding and binding pathway is encoded in the IDP itself; the reaction is not templated by the folded partner. We suggest that, by encoding both its transition state and level of residual structure, an IDP can evolve a specific kinetic profile, which could be a crucial functional advantage of disorder.

## Introduction

Folded proteins contain a plethora of information; three-dimensional conformations, folding pathways, (un)folding rates, stability, and function are all encoded within the amino acid chain (1). Unlike their folded counterparts, the primary sequences of intrinsically disordered proteins (IDPs)^5^ encode a *lack* of well-defined three-dimensional structure (2). Instead, they exist as an ensemble of conformations. When binding to a partner macromolecule, a subset of IDPs can transition from this conformational ensemble to a stable, well-defined structure (3, 4). Final conformations of IDPs in these folding-upon-binding reactions can differ depending on the partner protein. For example, the disordered C-terminal domain of p53 can fold and bind as a strand (5), a helix (6), or a coil (7) when interacting with Sir2, S100B(ββ), and cyclin A, respectively. In other cases, an IDP can form essentially the same structure when it binds promiscuously to a number of different partners, as when disordered BH3-only proteins bind to BCL-2 family proteins to form a single helix (8). The bound conformation may be transiently sampled by the free IDP but only becomes significantly populated in the presence of the stabilizing partner macromolecule. There are two extreme possibilities for the folding of the IDP in this situation. Either the IDP could encode the folding pathway (that is, determine the order of events), or the reaction could be templated by the partner protein (9).

Φ Value analysis is a method that probes the folding pathway by comparing the interactions a residue makes in the reaction transition state and the final, bound state (10). This is achieved through shortening the side chain of the amino acid, thus deleting contacts formed, and monitoring the impact on the stability and kinetics of the reaction. This method has been a useful tool, enabling the folding of families of homologous (well-folded) proteins to be compared (11), and it has also been used to investigate IDP–protein interactions (12–19). We previously used Φ value analysis to investigate the interaction between the intrinsically disordered BH3-only protein PUMA and the folded BCL-2–like protein MCL-1 (12). Here we investigate the binding of PUMA to A1, another folded BCL-2–like protein, and show that the pathway for folding and binding is conserved. Based on this observation, we hypothesized that the folding and binding pathway is encoded within the IDP sequence, and we performed a Φ value analysis for BID, another disordered BH3-only protein, when folding and binding to A1 and MCL-1 to test our assumptions. As predicted, BID displayed a conserved pattern of Φ values when binding to A1 and MCL-1, which differed from the pattern observed for PUMA. We therefore conclude that the folding and binding pathways for intrinsically disordered BH3-only proteins are encoded within the sequence of the IDP, not templated by the partner protein.

## Results

### Comparison of MCL-1 and A1

**Figure 1.**
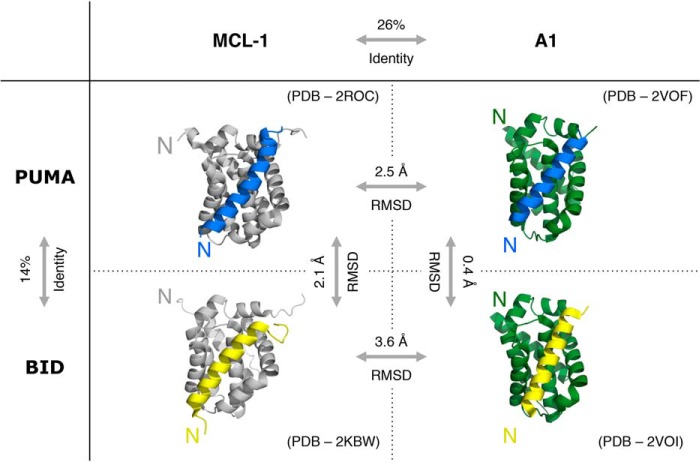
**Structural homology and sequence identity of complexes between BH3-only and BCL-2–like proteins.** The IDPs PUMA (*blue*) and BID (*yellow*) fold to a single contiguous α-helix upon binding either the folded partner protein MCL-1 (*gray*) or A1 (*green*). Sequence identities were produced from alignments of mouse protein constructs. Root mean square deviation (*RMSD*) values were obtained from backbone atom structural alignments using PyMOL. *N* indicates the N terminus of each protein.

**Figure 2.**
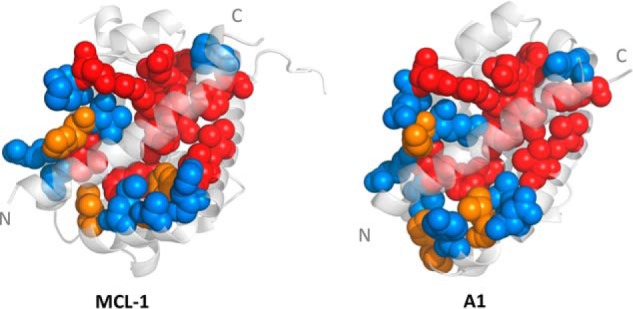
**The N-terminal region of PUMA contacts residues with different chemical properties when bound to MCL-1 and A1.** The bound structure of PUMA and noncontacting residues in MCL-1 and A1 are shown in *light gray*. Residues that contact PUMA were determined using PyMOL (PDB codes 2ROC and 2VOF), assuming the minimal cutoff (0.001 Å^2^) to account for all possible contacts between PUMA and its partners. Contacting residues are highlighted in the bound structure according to their conservation as follows: identical residues in *red*, similar in *orange* (*e.g.* both hydrophobic, same charges), and different in *blue* (*e.g.* opposite charges; one hydrophobic, one polar) (determined from the alignment of the MCL-1 and A1 sequences using Clustal Omega (32, 33)).

We have shown previously that PUMA has a relatively early transition state when folding and binding to MCL-1, with few inter- or intramolecular interactions formed (12). Residues toward the N terminus of PUMA display slightly higher Φ values, indicating that weak structure formation in this region stabilizes the transition state. To determine whether these transition state interactions are templated by the partner protein, we investigated the interaction of PUMA with A1, another folded BCL-2–like protein. Upon binding A1, PUMA folds into a single contiguous α-helix, forming a structure that closely resembles that of PUMA bound to MCL-1 (Fig. 1). Although the bound conformations are homologous, sequence alignment of the MCL-1 and A1 constructs showed that they share a total sequence identity of only 26% (Fig. 1 and Fig. S1*A*). Further analysis of the structures of PUMA bound to MCL-1 (20) and A1 (21) indicated that the binding interface shared a greater degree of identity (52%) because of a conserved seven-residue stretch (NWGRIVT). Six of these residues (NWGRVT) make contact with the C-terminal region of PUMA (Fig. 2 and Fig. S2). Importantly, the N-terminal region of PUMA, which had the highest Φ values when binding MCL-1 (12), contacts residues with different chemical properties in A1. Furthermore, the presence of Glu-78 and Glu-80 in A1 (Val-234 and Lys-236 in MCL-1) (Fig. S3) introduces a negative charge into the binding grove (21), whereas the electrostatic potential of the MCL-1 grove is positive (22). A1 therefore provides a good comparison with MCL-1, as PUMA folds to the same structure, but in the presence of different stabilizing amino acids with different physiochemical properties. If it were the binding partner that templates the folding pathway, then we might expect the pattern of Φ values to be different for PUMA binding to A1 and MCL-1.

### The folding pathway for the IDP PUMA is conserved when binding to different partners

**Figure 3.**
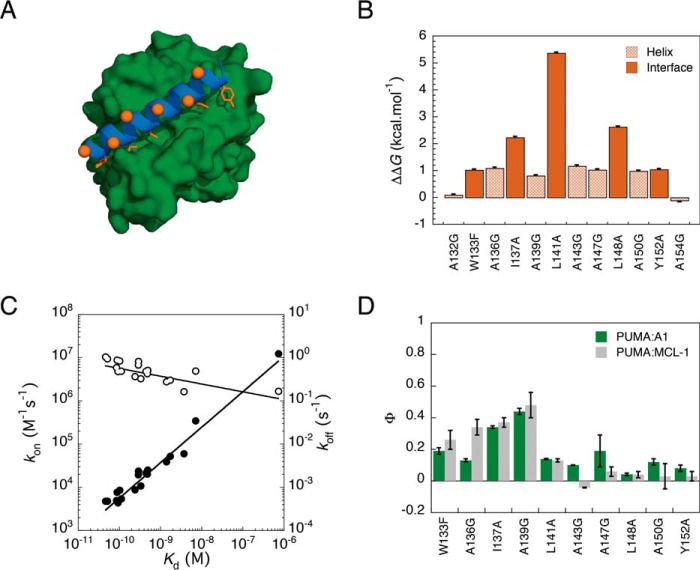
**Mutations to the IDP PUMA predominantly alter the affinity for A1 through modulating *k*_off_.** *A*, the position of PUMA (*blue*) mutations are indicated in *orange*. Mutations were either designed to probe interface interactions (*sticks*) with A1 (*green*) through shortening the side chain to alanine or intramolecular helicity (*spheres*) via alanine-to-glycine mutations. The N terminus of PUMA is shown on the *left. B*, binding destabilization upon probing PUMA interface interactions and helicity by mutation. *C*, linear free energy plot showing *k*_on_ (*open circles*) and *k*_off_ (*filled circles*) against *K_d_* for every PUMA mutant binding A1. *K_d_* values were calculated from the ratio of the kinetic rate constants (*k*_off_/*k*_on_), as the affinity was generally too tight (<1 nm) to measure using equilibrium binding experiments. Some mutations shifted the affinity to a regime that could be measured at equilibrium (Fig. S7). In these cases, the determined *K_d_* values compared well with the values calculated from the ratio of the rate constants (Table S4). *D*, comparison of Φ values obtained for PUMA binding to its folded partners, A1 and MCL-1. *Error bars* represent propagated errors.

As for the previous Φ value analysis (12), PUMA mutations were chosen that probed both the interfacial contacts and the amount of helicity in the reaction transition state (Fig. 3*A*). Binding was monitored by following the change in fluorescence of TAMRA, a fluorescent dye that was conjugated to the N terminus of PUMA, upon binding to A1 (Fig. S4). Ten of the 12 mutations resulted in a change in Gibbs free energy of binding of >0.6 kcal mol^−1^ (Fig. 3*B*), which is typically considered the minimum required to calculate Φ values (23). Analysis of all alanine and glycine mutants demonstrated that changes in *k*_off_, rather than *k*_on_, were predominantly responsible for differences in binding affinity (Fig. 3*C* and Table S1). From the linear free energy plot, the gradient of ln(*k*_on_) *versus* ln(*K_d_*) provided a Leffler α value of 0.18 ± 0.04, indicating that the transition state was structurally early. However, although this gives a global picture of the interactions formed in the transition state that is similar to PUMA binding MCL-1 (α = 0.1 ± 0.04) (12), it can hide the detail provided by a residue-level technique such as Φ value analysis. We therefore employed this method to determine which PUMA residues were forming interactions with A1 in the transition state. A remarkable similarity in Φ values was observed for PUMA binding A1 and PUMA binding MCL-1 (Fig. 3*D*). Both the pattern of higher Φ values at the N terminus and the absolute values were conserved when binding to either partner protein.

### Choice of mutations for the IDP BID

**Figure 4.**
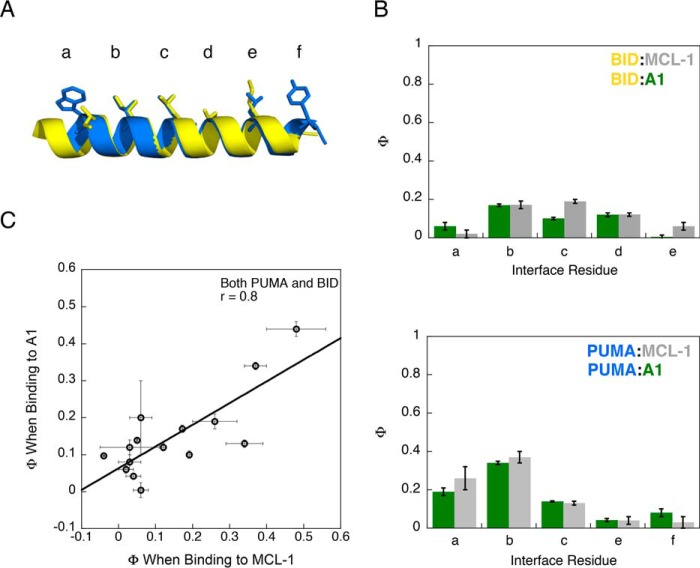
**The folding and binding pathway is encoded within the IDP rather than the stabilizing partner.** *A*, interface residues (*sticks*) in comparable helical turns were investigated for both the IDPs, PUMA (*blue*), and BID (*yellow*). The N terminus of each peptide is shown on the *left. B*, comparison of the Φ values obtained for the interface residues in BID (*top panel*) and PUMA (*bottom panel*) when binding to either A1 or MCL-1. *C*, correlation of Φ values obtained for the IDP when binding to MCL-1 and A1. Together with the IDP interface data, the Φ values from helix-probing mutations in PUMA that destabilized both the A1 and MCL-1 complex by >0.6 kcal mol^−1^ are included in the correlation plot. *Error bars* represent the propagated errors.

The resemblance of the reaction transition states indicated that the folding and binding pathway is encoded within the IDP (PUMA). To test the generality of this hypothesis, we investigated the binding of BID to A1 and MCL-1. The BH3-only region of BID is another intrinsically disordered protein (Fig. S5) that folds upon binding, forming a structure that is homologous to bound PUMA (Fig. 1). Because of the low sequence identity between BID and PUMA (14%) (Fig. S1*B*), the Φ values for BID should differ to those observed for PUMA if the IDP encodes the folding and binding pathway. BID residues that matched the position of the probed residues in PUMA were chosen for analysis. Five of the interface mutations (Fig. 4*A*) destabilized the complex with both A1 and MCL-1 by more than 0.6 kcal mol^−1^ (Tables S2 and S3) and were used to determine Φ values. Mutations designed to probe the transition state helicity of BID did not achieve this destabilization cutoff in both A1 and MCL-1 (Tables S2 and S3). A comparative analysis of the transition states of BID binding to A1 and MCL-1 was therefore produced using only interface Φ values.

### The folding and binding pathway is encoded within the IDP

All of the calculated interface Φ values were low (<0.2), indicating that few interactions were formed in the transition state. Unlike PUMA, which had higher Φ values at the N terminus, the pattern for BID indicated that interface interactions in the central section were stabilizing the transition state (Fig. 4*B*). The distinct pattern was replicated for BID binding to both A1 and MCL-1, supporting the original hypothesis that the transition state is encoded within the sequence of the IDP. When the data for both IDPs were combined, correlation of the Φ values obtained for the IDP with each partner protein (Fig. 4*C*) illustrated that the folding and binding information was contained within the IDP, not the partner protein (Fig. S6).

## Discussion

IDPs are typically overrepresented in cell signaling (24), a process that can comprise complex networks of many protein partners. The BCL-2 family is a model system for folding and binding reactions at the heart of these signaling networks. For example, PUMA and BID are disordered BH3-only proteins that can fold upon binding and initiate apoptosis through interacting with multiple partner proteins (25, 26).

Although Φ value analysis has been extensively used to characterize the folding pathway and mechanism of folded proteins, only a handful have been performed on other IDP-coupled folding and binding systems (12–19). Generally, these have demonstrated transition states that occur early along reaction coordinates, with few interactions formed (12–16). Our data for disordered BH3-only proteins binding BCL-2–like partners are consistent with this view of relatively unstructured transition states for IDPs that fold to simple conformations upon binding.

The N-terminal region of PUMA displays higher Φ values than the C-terminal end when binding to MCL-1, indicating that this region is important in stabilizing the reaction transition state (12). Given that IDPs can fold to different structures upon interacting with different partner proteins (5–7), it is easy to assume that the partner templates the folding reaction (9). If this is the case, then changing the partner protein to which PUMA should alter the transition state interactions. We therefore chose to investigate the binding of PUMA to a partner protein that had different interactions at the N terminus of PUMA and altered physiochemical properties of the binding groove (21, 22). In contrast to the templating hypothesis, we found that the transition state remained unchanged, indicating that the IDP (PUMA) encodes its transition state. This conclusion was supported by a Φ value analysis of the disordered BH3-only protein BID, which displayed a different pattern of Φ value than PUMA that was replicated when binding to either MCL-1 or A1. Interestingly, simulations of the unbound structure of PUMA indicate that it displays a greater degree of helicity toward the N terminus (27), whereas the CD analysis in this work indicated that BID is largely disordered when unbound. This is consistent with both transition states; PUMA has a slightly later transition state with higher Φ values at the N terminus, perhaps suggesting that the unbound structure of the IDP has an influence on the transition state interactions.

IDPs have the potential to encode both their level of residual structure and, as we show here, the structure of the transition states for coupled folding and binding; this provides an opportunity to evolve specific kinetic profiles. This could be of crucial importance in cell signaling processes, where disorder is conserved and abundant (24, 28), as responses to stimuli may have to occur quickly (*e.g.* activation of a cell surface receptor), or may need to be decisive and relatively irreversible (*e.g.* stimulation of apoptosis). Changing the residual structure or encoded transition state provides an accessible method for evolution to tune the lifetimes of these complexes, which may be one explanation for the evolutionary conservation of disorder.

## Experimental procedures

### Buffers

All biophysical experiments were carried out in 50 mm sodium phosphate, 0.05% (v/v) Tween 20 (pH 7.0).

### Proteins and peptides

BID (mouse, residues 76–110, UniProt P70444) and PUMA (mouse, residues 127–161, UniProt Q99ML1) peptides were synthesized by Biomatik. To reduce the oligomerization propensity, WT PUMA contained an M144A mutation, as described previously (29). A fluorescent dye, 5-carboxytetramethylrhodamine (TAMRA), was conjugated at the N terminus. The peptides were reconstituted in biophysical buffer and filtered (0.22 μm). Stock solutions were frozen in liquid N_2_ and stored at −80 °C. Expression and purification protocols for recombinantly produced MCL-1 (mouse, 152–308 residues, UniProt P97287), A1 (mouse, residues 1–152, UniProt Q07440), and PUMA are described in the supplemental Experimental Procedures.

### Circular dichroism

CD scans were acquired in an Applied Photophysics Chirascan using 1 cm (for PUMA) and 2 mm (for BID) path length cuvettes. Estimates for percentage helicity were calculated using the mean ellipticity at 222 nm and the method of Muñoz and Serrano (30). Scans were performed at multiple concentrations to check for the presence of oligomers (PUMA, 0.25–1 μm; BID, 5 and 10 μm).

### Binding kinetics

Association kinetics were monitored using SX18 and SX20 fluorescence stopped-flow spectrometers (Applied Photophysics) by following the TAMRA dye fluorescence, with excitation at 555 nm and emission recorded above 570 nm. Experiments were done at 25 °C, and a minimum of 15 fluorescence traces were collected and averaged before analysis. Data collected before the deadtime of mixing (1 ms) were removed. Pseudo-first-order conditions were adopted, with the concentration of the folded partner at least 10-fold higher than that of the peptide (31). For each peptide concentration, the traces were averaged and fit to a single exponential equation to extract the observed rate constant (*k*_obs_) of reaching the new equilibrium. The gradient from the straight line fit of *k*_obs_ *versus* the concentration of partner protein was used to determine *k*_on_.

Dissociation kinetics were monitored either using Applied Photophysics SX18 and SX20 stopped-flow spectrometers (*k*_obs_ > 0.03 s^−1^) or a Varian Cary Eclipse spectrophotometer (*k*_obs_ < 0.03 s^−1^) at 25 °C. A preformed complex of peptide and partner protein (PUMA–A1, BID–MCL-1, and BID–A1) was mixed with various concentrations of unlabeled PUMA peptide (used as a competitor). The change in fluorescence upon formation of the new equilibrium was monitored and fit to a single exponential function to determine *k*_obs_. With sufficient excess of the competitor, *k*_obs_ represents *k*_off_, and the concentration independent rate constants were averaged to ascertain *k*_off._

### Equilibrium binding

Equilibrium dissociation constants (*K_d_*) were measured at 25 °C by fluorescence anisotropy using a Cary Eclipse spectrophotometer (Varian). The TAMRA fluorophore was excited at 555 nm, and the emission was recorded at 575 nm. A detailed description of the data analysis used to extract the *K_d_* is included in the supplemental Experimental procedures.

### Data analysis

Data were analyzed using Kaleidagraph. The figures were prepared using Kaleidagraph and PyMOL. Errors were propagated using standard methods.

## Author contributions

M. D. C. and J. C. conceptualization; M. D. C., C. A. T. F. M., and Q. R. B. data curation; M. D. C., C. A. T. F. M., and Q. R. B. formal analysis; M. D. C. methodology; M. D. C., C. A. T. F. M., and J. C. writing-original draft; M. D. C., C. A. T. F. M., Q. R. B., and J. C. writing-review and editing; J. C. supervision; J. C. funding acquisition; J. C. project administration.

## Supplementary Material

Supporting Information

## Footnotes

5 The abbreviations used are:

IDP: intrinsically disordered protein
TAMRA: 5-carboxytetramethylrhodamine.
