# Supplementary material for: Folding and binding pathways of BH3-only proteins are encoded within their intrinsically disordered sequence, not templated by partner proteins
Source: J Biol Chem. 2018 May 1;293(25):9718–23. doi: 10.1074/jbc.RA118.002791 (PMC6016464; doi:10.1074/jbc.RA118.002791)
Supplement: Supporting Information [file supp_293_25_9718__index.html]

Folding and binding pathways of BH3-only proteins are encoded within their intrinsically disordered sequence, not templated by partner proteins — Encoding of Folding and Binding Pathways in IDPs — Folding and binding pathways of BH3-only proteins are encoded within their intrinsically disordered sequence, not templated by partner proteins — Encoding of folding and binding pathways in IDPs — Supporting Information 

# Folding and binding pathways of BH3-only proteins are encoded within their intrinsically disordered sequence, not templated by partner proteins

## Supporting Information

- Supporting Information for folding and binding pathways of BH3-only proteins are encoded within their intrinsically disordered sequence, not templated by partner proteins - Supporting Information
