# Supplementary material for: Folding and binding pathways of BH3-only proteins are encoded within their intrinsically disordered sequence, not templated by partner proteins
Source: J Biol Chem. 2018 May 1;293(25):9718–23. doi: 10.1074/jbc.RA118.002791 (PMC6016464; doi:10.1074/jbc.RA118.002791)
Supplement: Supporting Information [file supp_RA118.002791_136519_1_supp_126576_p7p4ww.pdf]

Supporting Information for

Folding and binding pathways of BH3-only proteins are encoded within their intrinsically disordered sequence, not templated by partner proteins

Michael D. Crabtree, Carolina A. T. F. Mendonça, Quenton R. Bubb, Jane Clarke

Correspondence to: jc162@cam.ac.uk

This PDF file includes:

SI Methods

Figures S1 to S7

Tables S1 to S4

SI References

SI Methods

Protein Expression and Purification. PUMA peptide (mouse, 127-161 residues, Uniprot Q99ML1), containing the mutation M144A, was used as wild type. The expression and purification was carried out as previously described (1) except for two nuances. Firstly, after binding to the Ni^{+2} agarose resin, the resin was washed twice with PBS 25 mM imidazole and secondly, Factor Xa cleavage was carried out in 20 mM Tris pH 8.0, 50 mM NaCl, 5 mM $CaCl_2$. Peptides were frozen using liquid N_2 and stored at -80°C.

MCL-1 gene (mouse, 152-308 residues, Uniprot P97287) was previously cloned into a modified version of the pRSET A vector, which contained a N-terminal hexahistidine tag and a thrombin cleavage site (2). In addition to the 157-amino acid construct, a GS remnant from thrombin cleavage was present at the N-terminus of MCL-1. The protein was expressed in *Escherichia coli* C41 pLysS cells and grown in LB media at 37°C. Once the optical density value at 600 nm reached 0.4-0.6 AU, expression was induced with 1 mM IPTG and cells were grown overnight at 18 °C. The cells were centrifuged, harvested, resuspended in PBS 25 mM imidazole and sonicated. Sonicate supernatant was incubated in Ni^{+2} agarose resin for 1 hour at 4°C. The resin was washed three times with PBS 25 mM imidazole to remove unbound protein. The bound protein was removed by elution with PBS 500 mM imidazole pH 7.5 and 4 mM of EDTA was added to the solution. The protein was buffer exchanged using 5 kDa molecular weight cut-off to reduce the imidazole concentration to ~ 50 mM. The HisTag was cleaved by incubating the solution with 600 units of thrombin overnight at room temperature. Uncleaved protein was removed from solution by incubation with Ni^{+2} agarose resin (4 mM of $CaCl_2$ was added to sequester the

free EDTA). MCL-1 was purified by ion-exchange followed by a size exclusion gel filtration step. Cleaved protein was loaded into a 5 mL HiTrap™ SP HP ion-exchange column using an AKTA FPLC. 10 mM HEPES pH 7.5 (with 0 or 1 M NaCl) buffers were used as ion-exchange buffers. Buffer gradients of 0 – 9% over 20 mL, 9 – 13% over 25 mL and 13 – 20% over 20 mL of NaCl were used to elute MCL-1. Pure MCL-1 (assessed by SDS-PAGE) was loaded into a Superdex G75 gel filtration column, which had been pre-equilibrated in 50 mM biophysical sodium phosphate buffer. MCL-1 was either stored at 4 °C or lyophilized after buffer exchanging into ddH_2O (HiTrap desalting columns).

A1 protein (mouse, residues 1-152, Uniprot Q07440), containing P104K and C113S mutations, was used as wild type. The gene was inserted into a modified pGEX-4T-3 vector that contained a tobacco etch virus (TEV) cleavage site. TEV cleavage results in an additional GS at the N-terminus of A1. A1 was expressed as it was previously described for MCL-1 (see above) except that after induction with IPTG, the overnight temperature was 24°C. After centrifugation and harvesting, the cells were resuspended in PBS and sonicated. A1 was expressed as a GST-fusion protein, hence the sonicate supernatant was incubated with glutathione sepharose 4B for four hours at 4°C. The bound protein was washed once with PBS and twice with 10 mM TRIS pH 8.0, 150 mM NaCl (TEV cleavage buffer). After resuspending in TEV cleavage buffer, EDTA and DTT were added to a final concentration of 2 mM and 10 mM, respectively. The solution was incubated overnight at room temperature with 5 µM of TEV protease. A1 was purified by ion-exchange followed by a size exclusion gel filtration step. Cleaved A1 was loaded into a 5 mL HiTrap™ SP HP ion-exchange column using an AKTA FPLC. 20 mM TRIS pH 7.0 (with 0 or 1 M

NaCl) were used as ion-exchange buffers. A buffer gradient of 0 – 23% NaCl over 60 mLs was used to elute A1. Pure A1 (assessed by SDS-PAGE) was loaded into a Superdex G75 gel filtration column as a final purification step and to exchange the buffer into the 50 mM biophysical sodium phosphate buffer. Purified A1 was stored at 4°C.

Protein and Peptide Concentration Measurements. Amino acid analysis provided an accurate extinction coefficient for the proteins: 7113 $M.cm^{-1}$ for PUMA (3), 22157 $M^{-1}.cm^{-1}$ for MCL-1 (2) and 24200 $M^{-1}.cm^{-1}$ for A1. Purified protein concentrations were measured by absorbance at 280 nm and their identities were confirmed using mass spectroscopy.

Accurate extinction coefficients for wild type labeled peptides were obtained by amino acid analysis (83000 $M^{-1}.cm^{-1}$ for both PUMA and BID). Labeled peptide concentrations were measured by absorbance at 555 nm (TAMRA dye).

Equilibrium Binding. The fluorescence intensity in vertically and horizontally polarized channels was used to calculate the anisotropy. A correction factor (*G* factor), was used to take into account the ability of the fluorimeter to detect vertically and horizontally polarized light. The *G* factor was calculated using equation 1:

$$G = \frac{I_{\mathrm{HV}}}{I_{\mathrm{HH}}} \tag{1}$$

where I_{HH} is the fluorescence intensity when the polarized light is excited and emitted horizontally and I_{HV} is the polarized light is excited horizontally and emitted vertically. Accounting for the G factor, the anisotropy (R) can be calculated by equation 2:

$$R = \frac{I_{VV} - GI_{VH}}{I_{VV} + 2GI_{VH}} \quad (2)$$

where I_{VV} is the fluorescence intensity when the excited and emitted light is vertically polarized and I_{VH} is the when the excitation light is vertically polarized and emitted and emission light is horizontally polarized.

To account for a change in fluorescence intensity of the dye upon binding (4), the anisotropy values were adjusted using equation 3:

$$R_{adj} = \frac{\frac{x}{y} \times \frac{Q_f}{Q_b} \times R_b + R_f}{1 + \frac{x}{y} \times \frac{Q_f}{Q_b}} \quad (3)$$

Where R_{adj} is the adjusted anisotropy, Q is the measured fluorescence intensity, R is the measured anisotropy, x is the difference between the measured anisotropy and the anisotropy of the free peptide and y is the difference between the anisotropy of the bound peptide and the measured anisotropy. The subscripts f and b indicate that the value is measured for the free or bound peptide, respectively. The fluorescence intensity of the free (Q_f) and bound (Q_b) peptide was calculated using equation 4:

$$Q = I_{\mathrm{VV}} + 2I_{\mathrm{VH}} \tag{4}$$

An estimate of the equilibrium dissociation constant (K_d) was then obtained by fitting each binding isotherm to equation 5:

$$R_{\mathrm{adj}} = R_{\mathrm{f}} + \Delta R\left(\frac{K_\mathrm{d}+[A]+[B]-\sqrt{((K_\mathrm{d}+[A]+[B])^2-4[A][B])}}{2[A]}\right) \tag{5}$$

where [A] is the concentration of dye-labelled peptide, [B] is the concentration of partner protein, R_f is the anisotropy of the free peptide and ΔR is the difference between the free and bound peptide anisotropies.

Φ-value calculations. Φ-values were calculated using equation 6:

$$\Phi = \frac{\ln\left(k_{\mathrm{on}}^{\mathrm{WT}}/k_{\mathrm{on}}^{\mathrm{MUT}}\right)}{\ln\left(K_{\mathrm{d}}^{\mathrm{MUT}}/K_{\mathrm{d}}^{\mathrm{WT}}\right)} \tag{6}$$

The K_d determined from equilibrium and kinetic studies showed good agreement for all for systems (PUMA-MCL-1, (3) PUMA-A1, BID-MCL-1 and BID-A1) (Fig. S7, Table S4), indicating that the reactions were two-state. We therefore typically calculated the K_d using equation 7:

$$K_\mathrm{d} = \frac{k_{\mathrm{off}}}{k_{\mathrm{on}}} \tag{7}$$

A

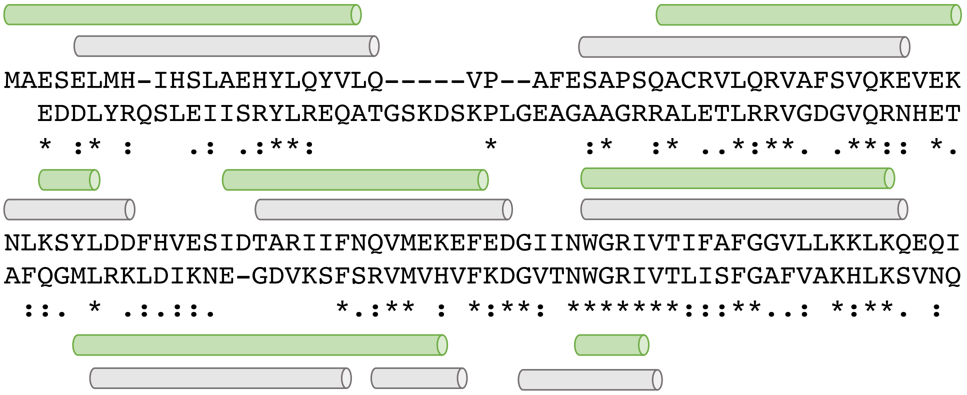

```
A1          MAESELMH-IHSLAEHYLQYVLQ-----VP--AFESAPSQACRVLQRVAFSVQKEVEK
MCL-1         EDDLYRQSLEIISRYLREQATGSKDSKPLGEAGAAGRRALETLRRVGDGVQRNHET
              * :* :   .: .:**:          *     :*  :* ..*:**. .**:: *.

A1          NLKSYLDDFHVESIDTARIIFNQVMEKEFEDGIINWGRIVTIFAFGGVLLKKLKQEQI
MCL-1       AFQGMLRKLDIKNE-GDVKSFSRVMVHVFKDGVTNWGRIVTLISFGAFVAKHLKSVNQ
             ::. * .:.::.       *.:** : *:**: *******:::**..: *:**. :

A1          ALDVSAYKQVSSFVAEFIMNNTGEWIRQNGGWEDGFIKKFEPKS----
MCL-1       ES---FIEPLAETITDVLVRTKRDWLVKQRGWD-GFVEFFHVQDLEGG
                   : ::. :::.::... :*: :: **: **:: *. :.
```

B

```
PUMA        RVEEEEWAREIGAQLRRAADDLNAQYERRRQEEQH
BID         SESQEEIIHNIARHLAQIGDEMDHNIQPTLVRQLA
              .:**  ::*. :* : .*::: : :    .:
```

Figure S1 Sequence alignments for the Al, MCL-1, PUMA and BID protein constructs. (A) Sequence alignment for A1 and MCL-1 with helical regions represented in green and grey, respectively. (B) Sequence alignment for PUMA and BID peptides. Alignments were produced using Clustal Omega (5, 6) (conserved residues are indicated with an asterisk, residues with strongly similar properties with a colon, and residues with weakly similar properties with a period). Note that in addition to the sequences shown above, both MCL-1 and A1 contained a GS at the N-terminus, which was a remnant from protease cleavage during purification.

```
A1          MAESELMH-IHSLAEHYLQYVLQ-----VP--AFESAPSQACRVLQRVAFSVQKEVEKNL
MCL-1       --EDDLYRQSLEIISRYLREQATGSKDSKPLGEAGAAGRRALETLRRVGDGVQRNHETAF
              *.:* :   .: .:**:          *     :*  :* ..*:**. .**:: *. :

A1          KSYLDDFHVESIDTARIIFNQVMEKEFEDGIINWGRIVTIFAFGGVLLKKLKQEQIALDV
MCL-1       QGMLRKLDIKNE-GDVKSFSRVMVHVFKDGVTNWGRIVTLISFGAFVAKHLKSVNQES--
            :. * .:.::.       *.:** : *:**: *******:::**..: *:**. :

A1          SAYKQVSSFVAEFIMNNTGEWIRQNGGWEDGFIKKFEPKS----
MCL-1       -FIEPLAETITDVLVRTKRDWLVKQRGWD-GFVEFFHVQDLEGG
               : ::. :::.::... :*: :: **: **:: *. :.
```

Figure S2. Comparison of the amino acids in MCL-1 and A1 that make contact with PUMA. Contacting residues are highlighted in grey. Alignments were produced using Clustal Omega (5, 6) (conserved residues are indicated with an asterisk, residues with strongly similar properties with a colon, and residues with weakly similar properties with a period). Note that in addition to the sequences shown above, both MCL-1 and A1 contained a GS at the N-terminus, which was a remnant from protease cleavage during purification.

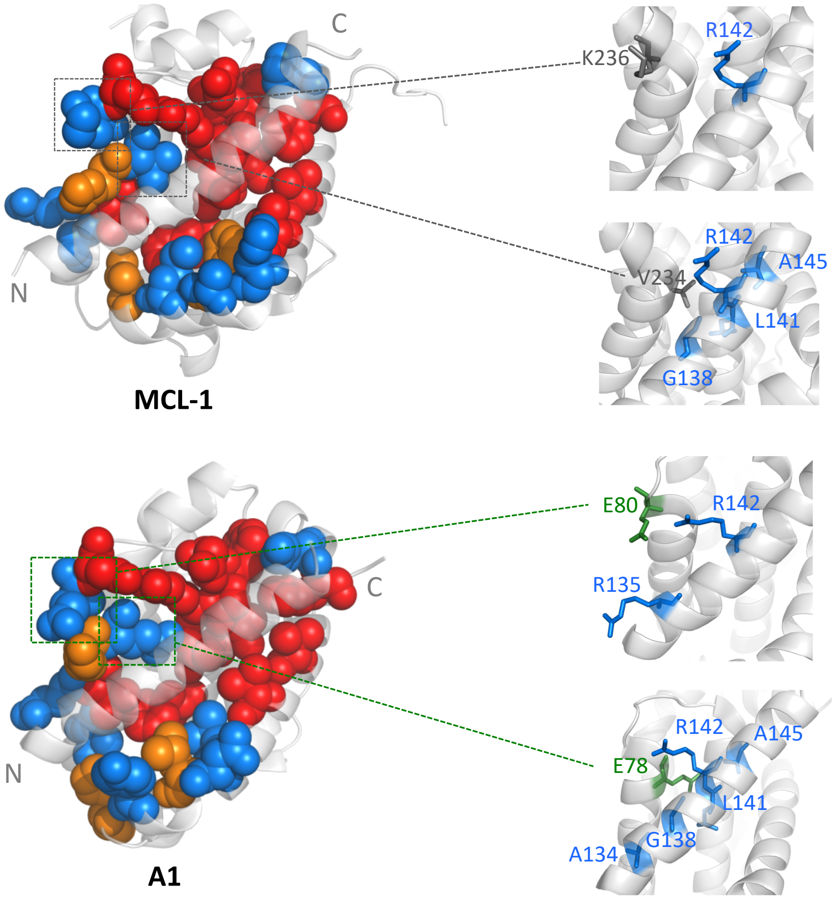

Figure S3. Lack of sequence conservation in the BCL-2-like protein binding grove results in differing interactions with BH3-only proteins. Comparison of the contacting residues in A1 and MCL-1 was performed as described in Figure 2 (identical residues in red; similar in orange; different in blue). The equivalent residues to V234 and K236 in MCL-1 (grey sticks) are E78 and E80 in A1 (green sticks). As well as altering the electrostatic properties of the binding grove, this results in differing interactions with PUMA (blue sticks). For example, the positively charged head group of PUMA R142 points towards the negatively charged E80 in A1, but away from the positively charged K236 in MCL-1. Residues in PUMA that contact A1 and MCL1 were determined using PyMOL (PDB code 2ROC and 2VOF), assuming the minimal cut-off (0.001 Å^2) to account for all possible contacts between PUMA and its partners.

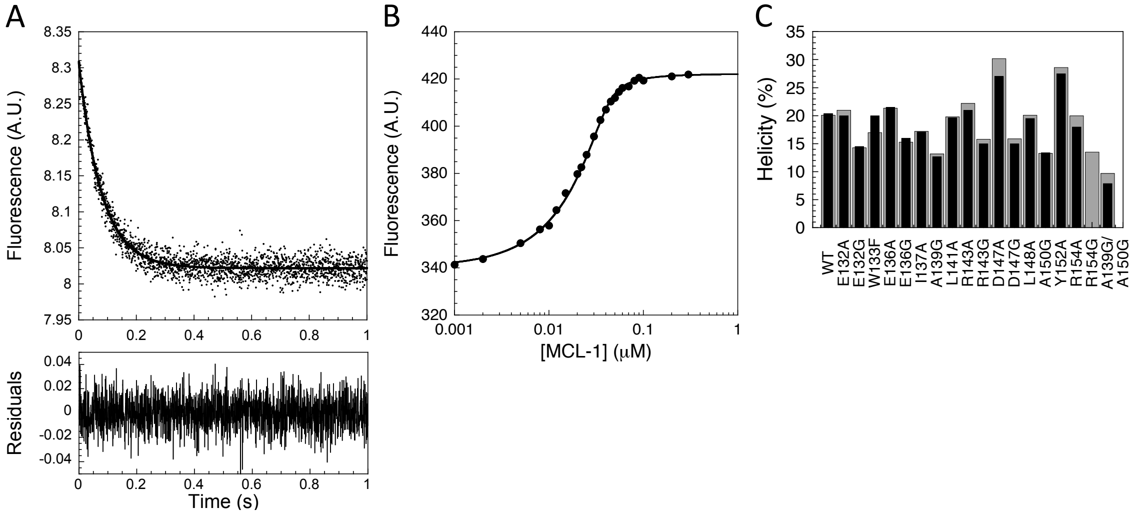

Figure S4. Addition of TAMRA dye does not change the interaction between PUMA and MCL-1. (A) Fluorescence signal of PUMA labeled with TAMRA dye shows a single exponential trace when binding to MCL-1, as for the unlabeled peptide (1). (B) Equilibrium binding curve for PUMA (A139G A150G) labeled with the TAMRA dye binding to MCL-1 shows identical K_d (1.7 ± 0.2 nM) when compared to the unlabeled PUMA (3). (C) Comparative helicity of PUMA peptides with (grey) and without (black) the TAMRA dye.

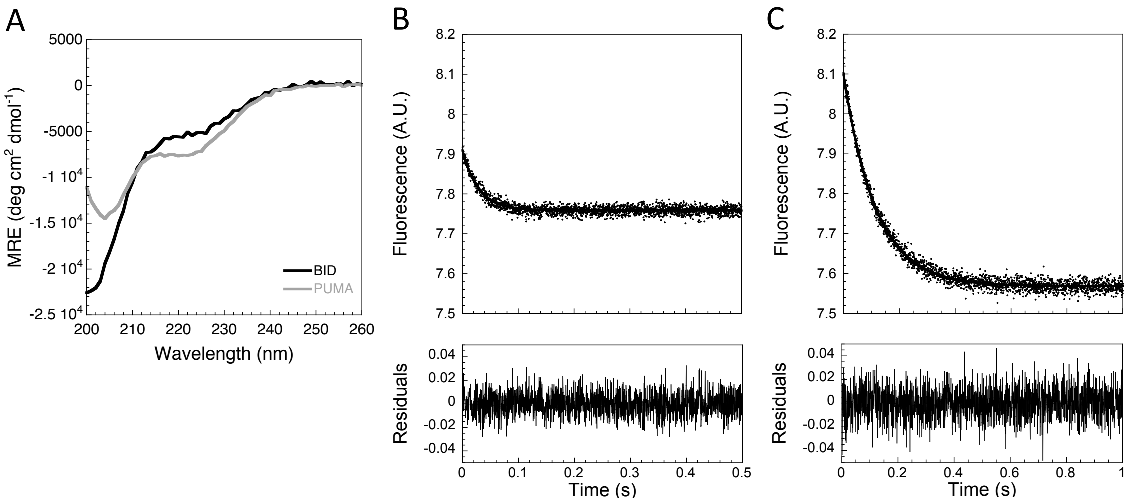

Figure S5. Characterization of the disordered peptide BID binding to MCL-1 and A1. (A) CD scans for BID and PUMA peptides, showing that BID is more disordered (less helical) than PUMA. (B & C) Examples of the change in fluorescence signal, fit to a single exponential, for TAMRA-labeled BID binding to (B) MCL-1 and to (C) A1.

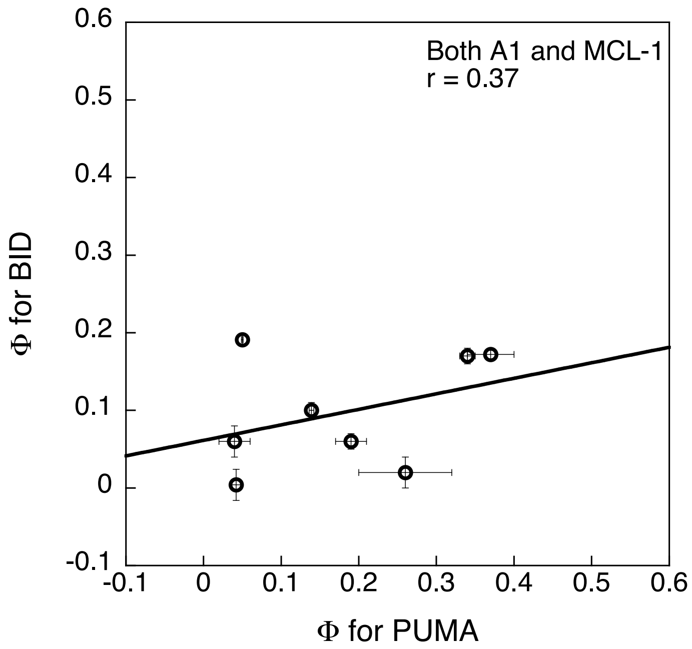

Figure S6. The binding partner does not template the coupled folding and binding reaction. Correlation plot of interface phi-values obtained for both partner proteins (MCL-1 and A1) when binding to the peptides PUMA and BID (helix-probing mutations did not destabilize the BID complexes enough to allow the calculation of a Φ-value). Error bars represent the propagated errors.

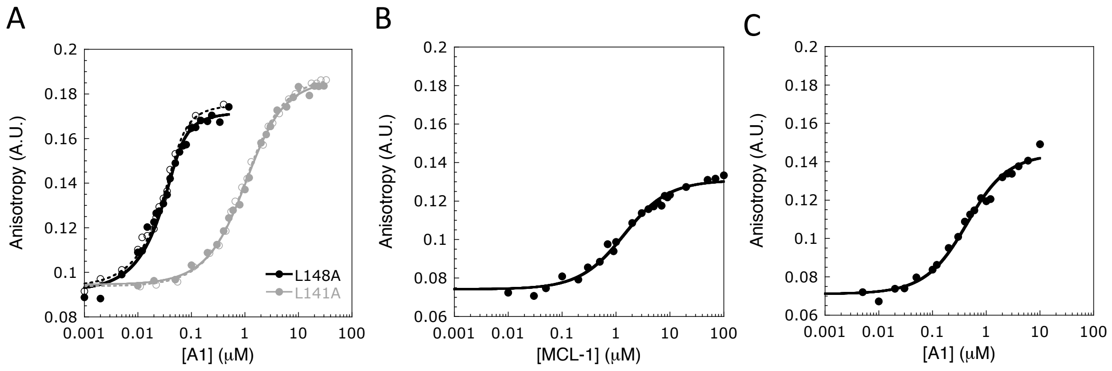

Figure S7. Equilibrium and kinetics K_d comparison show a two-state coupled folding binding reaction for PUMA-A1, BID-MCL-1 and BID-A1. (A) Equilibrium binding curves for PUMA L141A and L148A binding A1. Curve repeats are shown in open circles with the fit in dashed lines. (B) Equilibrium binding curve for BID-L90A binding MCL-1. (C) Equilibrium binding curve for BID-L90A binding A1. Comparison of the K_d values obtained by equilibrium binding and kinetics experiments are shown Table S4.

Table S1. Biophysical parameters for the coupled folding and binding of PUMA–A1.

PUMA	Helicity (CD) %	k_{on} ($\mu M^{-1}s^{-1}$)	k_{off} (s^{-1}) x10^{-3}	n	K_d (k_{off}/k_{on}) (nM)	$\Delta\Delta G$ (kcal.mol^{-1})	Φ
WT	20.1	5.9 ± 0.2	0.50 ± 0.02	4	0.085 ± 0.004	-	-
W133F	17.0	4.27 ± 0.04	2.03 ± 0.03	3	0.475 ± 0.008	1.02 ± 0.03	0.19 ± 0.02
I137A	17.2	1.64 ± 0.05	6.0 ± 0.2	4	3.7 ± 0.2	2.23 ± 0.04	0.34 ± 0.01
L141A	19.8	1.67 ± 0.01	1227 ± 8	4	735 ± 7	5.37 ± 0.03	0.139 ± 0.003
L148A	20.1	4.90 ± 0.05	34.8 ± 0.3	3	7.10 ± 0.09	2.62 ± 0.03	0.042 ±0.008
Y152A	28.6	5.1 ± 0.1	2.51 ± 0.03	3	0.49 ± 0.01	1.04 ± 0.03	0.08 ± 0.02
E132A	21.0	8.8 ± 0.5	0.77 ± 0.01	4	0.088 ± 0.005	0.02 ± 0.05	-
E132G	14.3	8.4 ± 0.2	0.86 ± 0.01	4	0.102 ± 0.003	0.09 ± 0.03	0.30 ± 0.02
E136A	21.4	10.4 ± 0.2	0.48 ± 0.04	4	0.046 ± 0.004	-0.36 ± 0.06	-
E136G	15.3	8.2 ± 0.2	2.38 ± 0.09	4	0.29 ± 0.01	1.09 ± 0.04	0.13 ± 0.01
A139G	13.2	3.26 ± 0.05	1.08 ± 0.04	4	0.33 ± 0.01	0.81 ± 0.04	0.44 ± 0.02
R143A	22.2	3.67 ± 0.04	0.88 ± 0.03	4	0.240 ± 0.008	0.62 ± 0.04	-
R143G	15.8	3.03 ± 0.01	5.2 ± 0.2	3	1.72 ± 0.07	1.17 ± 0.04	0.097 ± 0.002
D147A	30.2	9.4 ± 0.3	0.48 ± 0.02	4	0.051 ± 0.003	-0.92 ± 0.04	-
D147G	15.9	6.76 ± 0.08	1.95 ± 0.05	4	0.288 ± 0.008	1.03 ± 0.04	0.2 ± 0.1
A150G	13.3	4.84 ± 0.08	2.15 ± 0.02	4	0.444 ± 0.008	0.98 ± 0.03	0.12 ± 0.02
R154A	20.0	4.86 ± 0.08	0.54 ± 0.02	4	0.111 ± 0.005	0.16 ± 0.04	-
R154G	13.5	4.79 ± 0.03	0.44 ± 0.03	3	0.092 ± 0.006	-0.11 ± 0.05	-0.076 ± 0.002
A139G (A150G)	9.7	2.78 ± 0.02	3.9 ± 0.1	4	1.40 ± 0.04	0.68 ± 0.03	0.48 ± 0.01
A150G (A139G)	9.7	2.78 ± 0.02	3.9 ± 0.1	4	1.40 ± 0.04	0.82 ± 0.03	0.11 ± 0.01

Percentage of helicity was calculated using the MRE at 222 nm and the method of Munoz and Serrano (1995). k_{on} and k_{off} were experimentally determined, and the associated errors represent the curve fit error and the standard error of the mean (n= number of repeats used to determine k_{off}), respectively. K_d was calculated by taking the ratio between the dissociation and association rate constants, and the respective errors were propagated using standard methods. Φ was calculated using the kinetic rate constants and errors represent the propagated error.

Table S2. Biophysical parameters for the coupled folding and binding of BID–MCL-1.

BID	Helicity (CD) %	k_{on} ($\mu M^{-1}s^{-1}$)	k_{off} (s^{-1}) x10^{-3}	n	K_d (k_{off}/k_{on}) (nM)	$\Delta\Delta G$ (kcal.mol^{-1})	Φ
WT	11.5	8.9 ± 0.1	3.0 ± 0.1	4	0.33 ± 0.01	-	-
I82A	10.7	9.5 ± 0.9	6.20 ± 0.07	2	0.65 ± 0.06	0.40 ± 0.06	0 ± 300
I83A	12.0	8.6 ± 0.1	14 ± 4	4	1.7 ± 0.4	0.9 ± 0.1	0.02 ± 0.02
I86A	9.0	4.0 ± 0.1	140 ± 14	3	35 ± 4	2.78 ± 0.08	0.172 ± 0.008
L90A	7.5	2.1 ± 0.1	1300 ± 150	4	610 ± 80	4.50 ± 0.09	0.191 ± 0.008
I93A	8.9	4.5 ± 0.2	430 ± 26	4	96 ± 7	3.39 ± 0.06	0.121 ± 0.008
M97A	11.5	7.8 ± 0.3	30 ± 4	3	3.8± 0.5	1.47 ± 0.09	0.06 ± 0.02
I101A	10.1	8.2 ± 0.1	2.5 ± 0.2	4	0.30 ± 0.03	-0.06 ± 0.07	0 ± 1
I86A-M97A	9.0	3.5 ± 0.2	1360 ± 70	4	390 ± 30	4.23 ± 0.06	0.133 ± 0.007
E81A	10.0	6.5 ± 0.3	3.6 ± 0.3	4	0.55 ± 0.05	-	-
E81G	7.0	4.8 ± 0.1	8 ± 2	3	1.6 ± 0.3	0.6 ± 0.1	0.27 ± 0.06
R88A	11.3	10.0 ± 0.3	6.3 ± 0.7	3	0.6 ± 0.08	-	-
R88G	5.8	8.3 ± 0.5	15 ± 1	3	1.8 ± 0.2	0.6 ± 0.1	0.17 ± 0.06
E96A	13.0	9.9 ± 0.8	3.8 ± 0.6	4	0.38 ± 0.07	-	-
E96G	11.5	10.9 ± 0.4	10 ± 1	4	0.9 ± 0.1	0.5 ± 0.1	-0.1 ± 0.1
H99A	11.6	9.2 ± 0.4	1.0 ± 0.3	4	0.12 ± 0.03	-	-
H99G	13.0	9.0 ± 0.2	4.0 ± 0.1	4	0.4 ± 0.1	0.8 ± 0.2	0.01 ± 0.04

Percentage of helicity was calculated using the MRE at 222 nm and the method of Munoz and Serrano (1995). k_{on} and k_{off} were experimentally determined, and the associated errors represent the curve fit error and the standard error of the mean (n= number of repeats used to determine k_{off}), respectively. K_d was calculated by taking the ratio between the dissociation and association rate constants, and the respective errors were propagated using standard methods. Φ was calculated using the kinetic rate constants and errors represent the propagated error.

Table S3. Biophysical parameters for the coupled folding and binding of BID–A1.

BID	k_{on} ($\mu M^{-1}s^{-1}$)	k_{off} (s^{-1}) x10^{-3}	n	K_d (k_{off}/k_{on}) (nM)	$\Delta\Delta G$ (kcal.mol^{-1})	Φ
WT	8.2 ± 0.2	0.155 ± 0.005	3	0.019 ± 0.001	-	-
I82A	6.8 ± 0.2	0.300 ± 0.004	4	0.004 ± 0.001	0.49 ± 0.03	0.23 ± 0.04
I83A	7.0 ± 0.2	2.0 ± 0.5	4	0.29 ± 0.01	1.47 ± 0.02	0.06 ± 0.01
I86A	2.9 ± 0.1	22.0 ± 0.5	4	7.6 ± 0.2	3.55 ± 0.03	0.17 ± 0.01
L90A	3.1 ± 0.2	660 ± 3	5	210 ± 14	5.52 ± 0.04	0.10 ± 0.01
I93A	5.6 ± 0.1	2.5 ± 0.2	4	0.44 ± 0.03	1.87 ± 0.04	0.12 ± 0.01
M97A	8.1 ± 0.1	1.50 ± 0.04	4	0.190 ± 0.006	1.35 ± 0.03	0.004 ± 0.015
I101A	8.6 ± 0.3	0.20 ± 0.01	3	0.022 ± 0.001	0.10 ± 0.03	-0.3 ± 0.3
I86A- M97A	2.2 ± 0.1	110 ± 36	4	50 ± 16	4.650 ± 0.002	-
E81A	10.7 ± 0.5	0.213 ± 0.004	4	0.020 ± 0.001	-	-
E81G	9.6 ± 0.1	0.454 ± 0.001	4	0.047 ± 0.001	0.51 ± 0.03	0.12 ± 0.05
R88A	5.3 ± 0.2	0.88 ± 0.02	4	0.16 ± 0.08	-	-
R88G	4.9 ± 0.6	3.03 ± 0.06	4	0.6 ± 0.1	0.8 ± 0.3	0.06 ± 0.09
E96A	10 ± 2	0.146 ± 0.005	4	0.015 ± 0.002	-	-
E96G	7.8 ± 0.3	0.77 ± 0.02	4	0.098 ± 0.005	1.1 ± 0.1	0.13 ± 0.04
H99A	8.2 ± 0.2	0.182 ± 0.004	5	0.002 ± 0.001	-	-
H99G	8.2 ± 0.1	0.17 ± 0.01	4	0.002 ± 0.001	-0.04 ± 0.04	-0.03 ± 0.01

k_{on} and k_{off} were experimentally determined, and the associated errors represent the curve fit error and the standard error of the mean (n= number of repeats used to determine k_{off}), respectively. K_d was calculated by taking the ratio between the dissociation and association rate constants, and the respective errors were propagated using standard methods. Φ was calculated using the kinetic rate constants and errors represent the propagated error.

Table S4. Comparison between equilibrium and kinetics dissociation rate constants (K_d).

**Peptide**	**Partner Protein**	$K_{d\ (eq)}$ **(nM)**	$K_{d\ (kin)}$ (k_{off}/k_{on}) **(nM)**
PUMA L141A	A1	710 ± 20	735 ± 7
PUMA L148A	A1	5.65 ± 0.05	7.1 ± 0.1
BID L90A	MCL-1	1200 ± 140	610 ± 80
BID L90A	A1	360 ± 40	210 ± 14

$K_{d\ (eq)}$ was determined by equilibrium binding experiments following fluorescence anisotropy (see Methods for details). $K_{d\ (kin)}$ was calculated by taking the ratio between the dissociation and association rate constants. In both cases, the respective errors were propagated using standard methods.

References

1. Rogers, J. M., Wong, C. T., and Clarke, J. (2014) Coupled folding and binding of the disordered protein PUMA does not require particular residual structure. *J. Am. Chem. Soc.* **136**, 5197–5200
2. Rogers, J. M. J., Steward, A., and Clarke, J. (2013) Folding and binding of an intrinsically disordered protein: fast, but not "diffusion-limited". *J. Am. Chem. Soc.* **135**, 1415–1422
3. Rogers, J. M., Oleinikovas, V., Shammas, S. L., Wong, C. T., De Sancho, D., Baker, C. M., and Clarke, J. (2014) Interplay between partner and ligand facilitates the folding and binding of an intrinsically disordered protein. *Proc. Natl. Acad. Sci. U. S. A.* **111**, 15420–15425
4. Dandliker, W. B., Hsu, M. L., Levin, J., and Rao, B. R. (1981) Equilibrium and kinetic inhibition assays based upon fluorescence polarization. *Methods Enzymol.* **74**, 3–28
5. Goujon, M., McWilliam, H., Li, W., Valentin, F., Squizzato, S., Paern, J., and Lopez, R. (2010) A new bioinformatics analysis tools framework at EMBL-EBI. *Nucleic Acids Res.* **38**, W695–W699
6. Sievers, F., Wilm, A., Dineen, D., Gibson, T. J., Karplus, K., Li, W., Lopez, R., McWilliam, H., Remmert, M., Soding, J., Thompson, J. D., Higgins, D. G., Aniba, M., Poch, O., Thompson, J., Blackshields, G., Sievers, F., Shi, W., Wilm, A., Higgins, D., Bradley, R., Roberts, A., Smoot, M., Juvekar, S., Do, J., Dewey, C., Holmes, I., Pachter, L., Clamp, M., Cuff, J., Searle, S., Barton, G., Do, C., Mahabhashyam, M., Brudno, M., Batzoglou, S., Eddy, S., Edgar, R., Edgar, R., Finn, R., Mistry, J., Tate, J., Coggill, P., Heger, A., Pollington, J., Gavin, O., Gunasekaran, P., Ceric, G., Forslund, K., Holm, L., Sonnhammer, E., Eddy, S., Bateman, A., Gouy, M., Guindon, S., Gascuel, O., Hogeweg, P., Hesper, B., Katoh, K., Misawa, K., Kuma, K., Miyata, T., Katoh, K., Toh, H., Larkin, M., Blackshields, G., Brown, N., Chenna, R., McGettigan, P., McWilliam, H., Valentin, F., Wallace, I., Wilm, A., Lopez, R., Thompson, J., Gibson, T., Higgins, D., Lassmann, T., Sonnhammer, E., Liu, Y., Schmidt, B., Maskell, D., Löytynoja, A., Goldman, N., Mizuguchi, K., Deane, C., Blundell, T., Overington, J., Morgenstern, B., Frech, K., Dress, A., Werner, T., Notredame, C., Higgins, D., Heringa, J., Pirovano, W., Feenstra, K., Heringa, J., Söding, J., Thompson, J., Koehl, P., Ripp, R., Poch, O., Wilm, A., Higgins, D., and Notredame, C. (2014) Fast, scalable generation of high-quality protein multiple sequence alignments using Clustal Omega. *Mol. Syst. Biol.* **7**, 539–539
